# Intranodular and perinodular radiomics features based on non-contrast CT to distinguish pulmonary cryptococcosis from lung adenocarcinoma: a two-center study

**DOI:** 10.3389/fonc.2026.1750773

**Published:** 2026-04-27

**Authors:** Yanfang Deng, Lin Lin, Kaiji Deng, Liqing Xie, Faming Lai, Suhua Zhong, Yunshan Jiang, Yunjing Xue

**Affiliations:** 1 Department of Radiology, Longyan First Affiliated Hospital of Fujian Medical University, Longyan, Fujian, China; 2 Department of Radiology, Fujian Medical University Union Hospital, Fuzhou, Fujian, China; 3 Department of Oncology, Longyan First Affiliated Hospital of Fujian Medical University, Longyan, Fujian, China; 4 School of Medical Technology and Engineering, Fujian Medical University, Fuzhou, Fujian, China; 5 Fujian Key Laboratory of Intelligent Imaging and Precision Radiotherapy for Tumors (Fujian Medical University), Fuzhou, China

**Keywords:** computed tomography, lung adenocarcinoma, pulmonary cryptococcosis, radiomics, texture analysis

## Abstract

**Background:**

Distinguishing pulmonary cryptococcosis (PC) from lung adenocarcinoma (LAC) remains clinically challenging in practice. The purpose of this study was to investigate the utility of intranodular and perinodular radiomics features derived from non-contrast CT in differentiating PC from LAC.

**Materials and methods:**

A total of 244 patients with PC and LAC from two centers were randomly divided into a training set and a testing set at a ratio of 7:3. Logistic regression analysis was used to establish the clinical model. Radiomics features were extracted from the lesions and lesion margins of 10 mm. Support vector machine (SVM) was used to construct the intranodular, perinodular, and combined radiomics models. The areas under the receiver operating characteristic curve (AUCs) and decision curve analysis (DCA) were employed to assess the diagnostic performance, while the DeLong test was applied for model comparisons.

**Results:**

The three radiomics models exhibited excellent diagnostic performance for identifying PC and LAC, with the combined radiomics model achieving the highest AUC value in both the training (AUC = 0.936, sensitivity = 0.838, specificity = 0.898, accuracy = 0.859) and testing sets (AUC = 0.922, sensitivity = 0.854, specificity = 0.808, accuracy = 0.892). In the testing set, the AUC of the combined radiomics model was significantly higher than that of the clinical model (*p* = 0.005), while no statistically significant difference was found when compared with the intranodular or the perinodular model (*p* > 0.05). The combined model outperformed the other three models according to the DCA in terms of net benefit.

**Conclusion:**

A combined radiomics model integrating intranodular and perinodular features can effectively improve diagnostic accuracy in differentiating PC from LAC.

## Introduction

Pulmonary cryptococcosis (PC) is the second most prevalent invasive pulmonary fungal disease in China. Historically, it has been regarded as a condition predominantly affecting immunocompromised populations such as those with AIDS. Nonetheless, the incidence of PC has remained persistently high among immunocompetent individuals in recent years (1, 2). At present, it remains prevalent worldwide and represents a significant threat to human life and health.

Clinically, PC most frequently presents as nodular or mass lesions, typically accompanied by nonspecific clinical manifestations or unremarkable laboratory results (3). Standardized antifungal therapy enables remission or cure in the majority of PC cases. In contrast, lung adenocarcinoma (LAC) generally necessitates surgical intervention or neoadjuvant therapy. Notably, PC exhibits overlapping morphological features with lung cancer on computed tomography (CT) imaging, such as irregular shape, halo sign, lobulation, spiculation, and mediastinal lymph node enlargement (4). Furthermore, positron emission tomography (PET)-CT scans commonly show high fluorodeoxyglucose (FDG) uptake in both PC and LAC (5). This poses substantial diagnostic challenges and predisposes to misdiagnosis as LAC, particularly in cases of solitary nodules. This, in turn, increases the likelihood of unnecessary invasive diagnostic procedures or surgical resection. Hence, the development of a noninvasive diagnostic approach capable of accurately distinguishing between PC and LAC has become an immediate priority.

Radiomics, an emerging noninvasive quantitative analytical approach, facilitates the quantitative assessment of pulmonary nodule heterogeneity through the in-depth extraction of multidimensional imaging features (including textural, morphometric, and intensity-based characteristics) from medical images. These features exhibit strong correlations with biological behaviors and internal microstructure alterations, thereby enabling radiomics to serve as a foundational tool for personalized clinical decision-making across multiple domains, including clinical diagnosis, genotyping, and therapeutic response assessment (6–8). Several studies have established that radiomics models based on primary lesions can effectively differentiate lung cancer from inflammatory nodules, with favorable and robust predictive performance (9–13). For example, Yang et al. (9) established a radiomics model to differentiate tuberculosis granulomas from LACs with high accuracy. As reported by Adelsmayr et al. (13), significant differences in the imaging features between malignant lesions and organizing pneumonia can be detected via CT texture analysis. Previous studies have also demonstrated the potential of CT radiomics features extracted from lesions in differentiating PC from lung cancer (14, 15). Recent studies have revealed that the perinodular zone contains supplementary information, and the integration of radiomics features from both the perinodular and intranodular regions may provide incremental diagnostic value (16–18). Although radiomics has been used to differentiate PC from LAC, only a few studies have investigated the utility of perinodular features. Therefore, we hypothesized that perinodular radiomics features represent a promising yet underexplored avenue for improving the diagnostic accuracy in differentiating PC from LAC.

The present study was designed to develop and validate intranodular and perinodular radiomics features derived from non-enhanced CT for differentiating PC from LAC and to further establish an optimized model to improve diagnostic performance.

## Materials and methods

### Patients

This retrospective study was approved by Longyan First Affiliated Hospital of Fujian Medical University (center 1) and Fujian Medical University Union Hospital (center 2). The necessity for written informed consent was waived. Patients with PC or LAC between January 2015 and December 2024 were retrospectively identified from the electronic medical records of the two centers. The inclusion criteria were as follows: a) LAC and PC histopathologically confirmed by biopsy or surgical resection; b) solitary solid nodules or masses with a maximum diameter ≤3 cm; and c) non-contrast chest CT images with slice thickness ≤1.5 mm acquired within 2 weeks prior to pathological examination, with patients not having received relevant treatment before biopsy and resection. The exclusion criteria were as follows: a) PC complicated with other pathogenic infections or patients with LAC with a history of malignant tumors; b) poor image quality; and c) multiple lesions.

**Figure 1:**
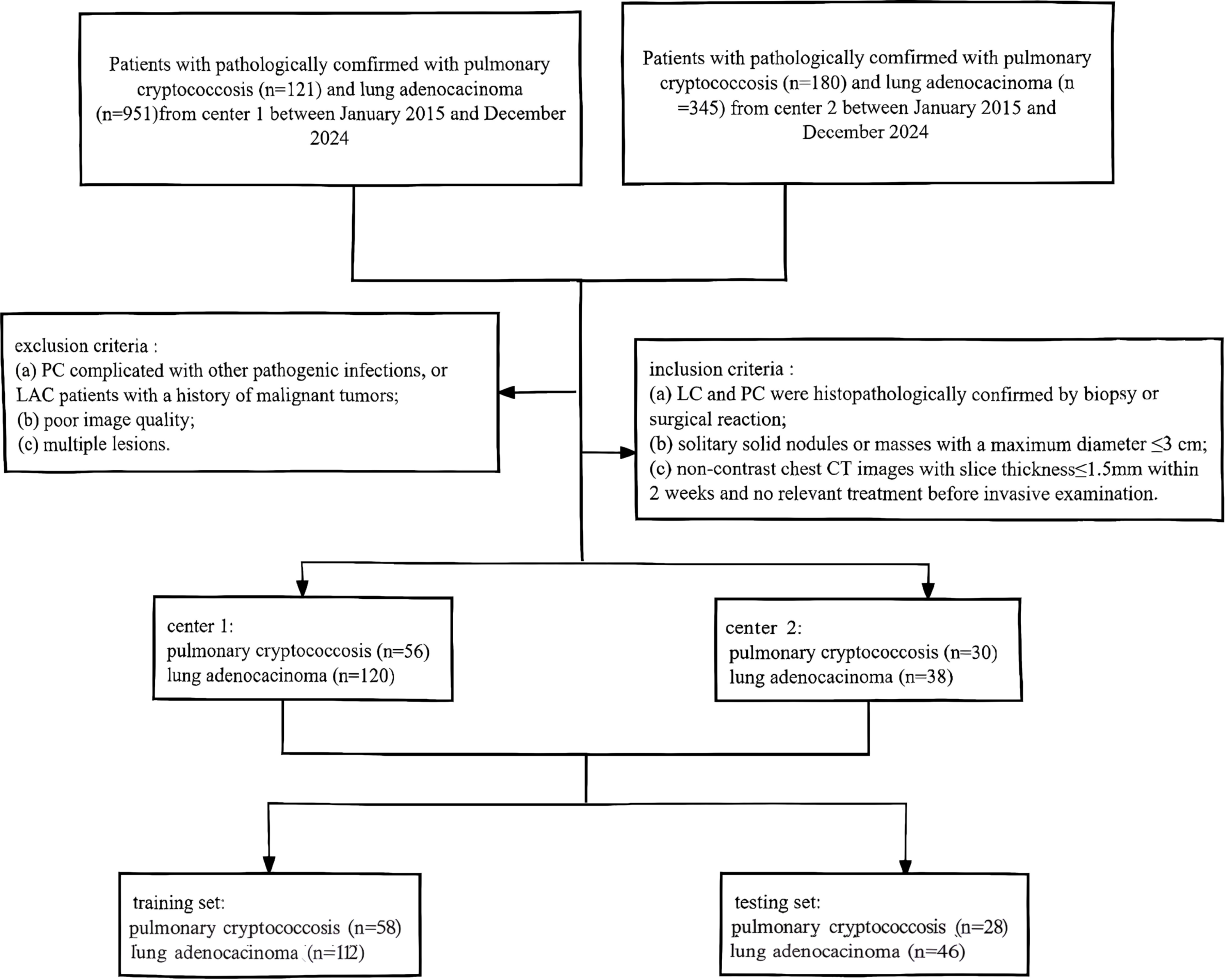
Flowchart of the inclusion and exclusion criteria for patient selection.

Finally, 176 patients (56 with PC and 120 with LAC) from center 1 and 68 patients (30 with PC and 38 with LAC) from center 2 were enrolled. Subsequently, all patients were randomly divided into a training set (112 LAC and 58 PC) and a testing set (46 LAC and 28 PC) at a ratio of 7:3. The sample size and distribution were highly comparable to those reported in a previous radiomics study (19). The study selection criteria are presented in a schematic illustration in **Figure 1**.

### CT image acquisition

All non-contrast chest CT scans were obtained from one of five CT scanners: GE Light Speed VCT, GE Discovery CT750 HD, GE Revolution 256 (GE HealthCare, Chicago, IL, USA), SOMATOM Force dual-source CT (Siemens Healthineers, Erlangen, Germany), and Philips Brilliance iCT 256 (Philips Medical Systems, Cleveland, OH, USA). The detailed scanning parameters were as follows: tube voltage of 120 keV, tube current of 280–320 mAs, pitch of 0.8–1.0, rotation time of 0.75 s, and matrix of 512 × 512 or 1,024 × 1,024. Reconstruction was performed using a standard algorithm with a reconstruction slice thickness and interval of 0.625–1.5 mm. Patients were positioned supine in a head-first position, with the scanning range extending from the lung apex to the lung base. All scans were acquired during end-inspiratory breath-hold.

### Evaluation of clinical and radiographic characteristics

Clinical data including gender, age, smoking history, and serum cytokeratin 19 fragment (CYFRA21-1) levels were collected. CYFRA21–1 levels exceeding 3.3 ng/ml were considered elevated. In addition, the radiographic features were independently analyzed by two experienced radiologists (each with more than 10 years of experience in chest imaging) who were blinded to the pathological results. The following radiological features were documented: lesion location, shape, maximum diameter, presence of lobulation, spiculation, vacuole signs, pleural indentation, and air bronchogram. Discrepancies were resolved by consensus.

### Image segmentation and feature extraction

**Figure 2:**
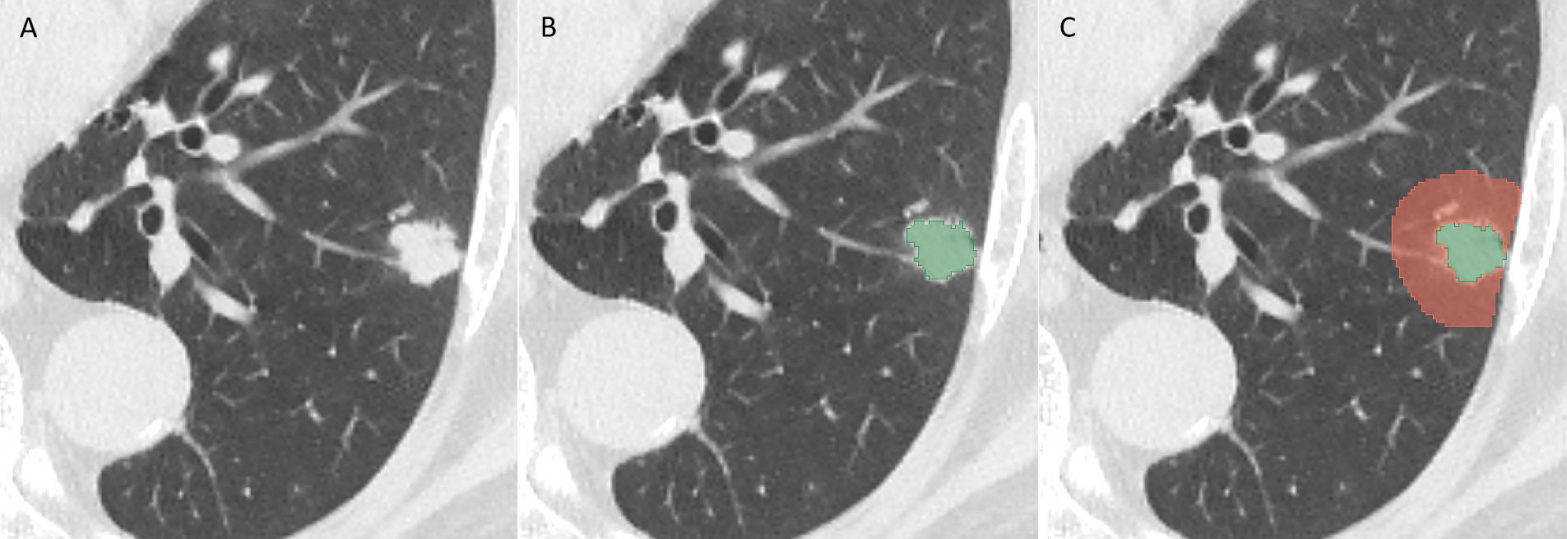
Volume of interest (VOI) segmentation of the lesions. **(A)** An irregular nodule on the non-enhanced CT image. **(B)** The lesion volume (*green region*) was manually delineated slice by slice on lung windows. **(C)** It was automatically expanded outward by 10 mm from the nodular edge, and the lesion components and structures such as bones were manually removed to obtain the perilesional volume (*red region*).

Image segmentation and feature extraction were performed using the open-source software 3D Slicer (version 5.0.2; http://www.slicer.org). A radiologist with 14 years of experience in diagnosing chest diseases manually delineated the three-dimensional volume of interest (VOI) of each nodule on the lung window (window width, 1200 HU; window level, −600 HU) using the Segment Editor module. The lesion volume (LV) was acquired, ensuring the exclusion of necrosis, large vessels, bronchi, and calcifications. Subsequently, the software automatically expanded the VOI to include the surrounding parenchymal tissue within a 10-mm margin, and the intranodular components and adjacent structures such as bones were removed from the expanded mask to obtain the perilesional volume (PLV), as shown in **Figure 2**. Furthermore, to assess inter-observer reproducibility, a secondary radiologist with 15 years of experience independently performed image segmentation on 40 randomly selected cases from the entire cohort. The intraclass correlation coefficient (ICC) was then calculated for the intranodular and perinodular radiomics features to evaluate the inter-observer reliability and the impact of segmentation variability on feature stability.

As a preprocessing step for feature extraction, all images were uniformly resampled to an isotropic voxel size of 1 mm × 1 mm × 1 mm and normalized using a bin width of 25 HU. In total, 1,130 radiomics features were extracted from each VOI from the original images, as well as those of the wavelet- and Laplacian of Gaussian (LOG)-filtered images (with sigma values of 1, 2, and 3). These features comprised seven categories: 14 shape features, 18 first-order features, 24 gray-level co-occurrence matrix (GLCM) features, 14 gray-level dependence matrix (GLDM) features, 16 gray-level size zone matrix (GLSZM) features, 16 gray-level run length matrix (GLRLM) features, and 5 neighborhood gray-tone difference matrix (NGTDM) features.

### Feature selection and model development

Data partitioning and all subsequent analyses were performed using the open-source software Feature Explorer (FAE, version 0.5.2) (20). The dataset was randomly divided into a training and a testing set, with center 1 contributing 69.3% (122/176) and 30.7% (54/176) and center 2 contributing 63.2% (43/68) and 36.8% (25/68) to the training and testing sets, respectively. Only those features with ICC > 0.75 were retained for feature selection and model development. In addition, upsampling was applied to address data imbalance. Thereafter, the features were standardized and normalized using the mean method. Given the high dimensionality of the feature space, Pearson’s correlation coefficient (PCC) analysis was performed to remove redundant features, where, if a pair exhibited high correlation (PCC > 0.9), one feature was eliminated. Following feature selection with recursive feature elimination (RFE), 1–15 optimal features were retained. Using a support vector machine (SVM) classifier with fivefold cross-validation, three radiomics models were constructed based on the intranodular, perinodular, and combined (intranodular + perinodular) features. Final model performance was assessed on the independent testing set, and the model that achieved the highest area under the receiver operating characteristic curve (AUC) was selected as the optimal model.

### Statistical analysis

Normally distributed quantitative data were presented as the mean ± standard deviation and were compared between groups using the independent *t*-test. Non-normally distributed data were presented as median and interquartile ranges (IQR) and were compared using the Mann–Whitney *U* test. Categorical variables were analyzed using the chi-square test or Fisher’s exact test and were reported as frequencies and percentages. In addition, prior to multivariate analysis, collinearity diagnostics were performed using linear regression analysis, and variance inflation factor (VIF) values were calculated. A VIF value greater than 5 was considered indicative of significant multicollinearity. Univariate and multivariate logistic regression analyses were performed to identify significant clinical and radiological characteristics, which were then used to construct a clinical model. ICC was applied for inter-observer consistency analysis, with an ICC > 0.75 indicating good agreement. Model performance was evaluated using AUC, with 95% confidence interval (CI), sensitivity, specificity, accuracy, positive predictive value (PPV), and negative predictive value (NPV). The optimal cutoff value for the calculation of these metrics was determined by maximizing the Youden index (Youden’s index = sensitivity + specificity − 1). Pairwise comparisons of the AUCs were performed using the DeLong test. Decision curve analysis (DCA) was then used to estimate the net benefit of the model. A two-sided *p*-value <0.05 was considered statistically significant. SPSS software (version 22.0) and MedCalc software (version 20.1.0) were used to perform statistical analysis. The DCA curves were plotted using R software (version 4.5.1; https://www.r-project.org/).

## Results

### Clinical and radiological characteristics

The clinical and radiological characteristics of the 244 patients from the training and testing sets are presented in **Table 1**. In the training set, statistically significant differences (all *p* < 0.05) in age, smoking history, lesion location, spiculation, pleural indentation, vacuole signs, and maximum diameter were identified between the PC and LAC groups. There were no significant differences in sex, CYFRA21-1, shape, lobulation, and air bronchogram between the two groups in the training set (all *p* > 0.05). However, in the testing set, statistically significant differences were only detected in age and pleural indentation between the two groups (*p* < 0.05). Linear regression analysis showed that all candidate variables had VIF values ranging from 1.1 to 1.6, indicating no significant collinearity among the predictors. Subsequently, the univariate and multivariate logistic regression analyses revealed that younger age (OR = 0.913, 95%CI = 0.866–0.962, *p* = 0.001) and the absence of pleural indentation (OR = 15.27, 95%CI = 5.218–44.685, *p* < 0.001) were identified as statistically significant independent protective factors for PC in the training set (**Table 2**). These two factors were used to construct the clinical model.

**Table 1:** Clinical and radiological characteristics of the patients in the training and testing sets.

Variable	Training set		*p*-value	Testing set		*p*-value
	LAC (*n* = 112)	PC (*n* = 58)		LAC (*n* = 46)	PC (*n* = 28)	
Sex			0.318			0.168
Male	65 (58%)	29 (50%)		22 (47.8%)	18 (64.3%)	
Female	47 (42%)	29 (50%)		24 (52.2%)	10 (35.7%)	
Age (years), mean ± SD	62.11 ± 9.75	50.36 ± 9.40	<0.001*	60.09 ± 8.99	52.61 ± 10.15	0.001*
Smoking history			0.005*			0.716
Yes	66 (58.9%)	47 (81%)		15 (32.6%)	8 (28.6%)	
No	46 (41.1%)	11 (19%)		31 (67.4%)	20 (71.4%)	
CYFRA21-1			0.09			0.372
Normal	84 (75%)	50 (86.2%)		37 (80.4%)	20 (71.4%)	
Elevated	28 (25%)	8 (13.8%)		9 (19.6%)	8 (28.6%)	
Location			0.042*			0.484
Upper lobe	63 (56.3%)	21 (36.2%)		27 (58.7%)	14(50%)	
Middle lobe	6 (5.4%)	3 (5.2%)		2 (4.3%)	3 (10.7%)	
Lower lobe	43 (38.4%)	34 (58.6%)		17 (37%)	11 (39.3%)	
Shape			0.544			0.896
Round/oval	71 (63.4%)	34 (58.6%)		19 (41.3%)	12 (42.9%)	
Irregular	41 (36.6%)	24 (41.4%)		27 (58.7%)	16 (57.1%)	
Lobulation			0.606			0.656
Yes	87 (77.7%)	43 (74.1%)		35 (76.1%)	20 (71.4%)	
No	25 (22.3%)	15 (25.9%)		11 (23.9%)	8 (28.6%)	
Spiculation			0.017*			0.304
Yes	66 (58.9%)	23 (39.7%)		27 (58.7%)	13 (46.4%)	
No	46 (41.1%)	35 (60.3%)		19 (41.3%)	15 (53.6%)	
Pleural indentation			<0.001*			0.014*
Yes	83 (74.1%)	7 (12.1%)		33 (71.7%)	12 (42.9%)	
No	29 (25.9%)	51 (87.9%)		13 (28.3%)	16 (57.1%)	
Air bronchogram			0.05			0.339
Yes	46 (41.1%)	15 (25.9%)		16 (34.8%)	6 (21.4%)	
No	66 (58.9%)	43 (25.9%)		30 (65.2%)	22 (78.6%)	
Vacuole sign			0.01*			0.499
Yes	28 (25%)	5 (8.6%)		9 (19.6%)	3 (10.7%)	
No	84 (75%)	53 (91.4%)		37 (80.4%)	25 (89.3%)	
Maximum diameter (cm)	1.9 (1.3–2.6)	1.5 (1.1–2.1)	0.021*	1.8 (1.4–2.8)	1.7 (1.3–2.3)	0.438

*LAC*, lung adenocarcinoma; *PC*, pulmonary cryptococcosis; *SD*, standard deviation; *CYFRA21-1*, cytokeratin 19 fragment; * Indicates p < 0.05.

**Table 2:** Multivariate logistic regression in the training set.

Variable	Univariate		Multivariate	
	OR (95%CI)	*p*-value	OR (95%CI)	*p*-value
Age	0.883 (0.846–0.921)	<0.001*	0.913 (0.866–0.962)	0.001*
Smoking history	2.978 (1.397–6.347)	0.005*	1.827 (0.644–5.18)	0.257
Location	1.596 (0.822–3.099)	0.167	–	–
Spiculation	2.183 (1.143–4.169)	0.018*	0.759 (0.288–2,001)	0.577
Pleural indentation	20.852 (8.512–51.084)	<0.001*	15.27 (5.218–44.685)	<0.001*
Vacuole sign	3.533 (1.285–9.718)	0.014*	1.084 (0.283–4.156)	0.906
Maximal diameter (cm)	0.0672 (0.451–1.002)	0.051	–	–

*OR*, odds ratio; *95%CI*, 95% confidence interval; * Indicates p < 0.05.

### Model performance

**Figure 3:**
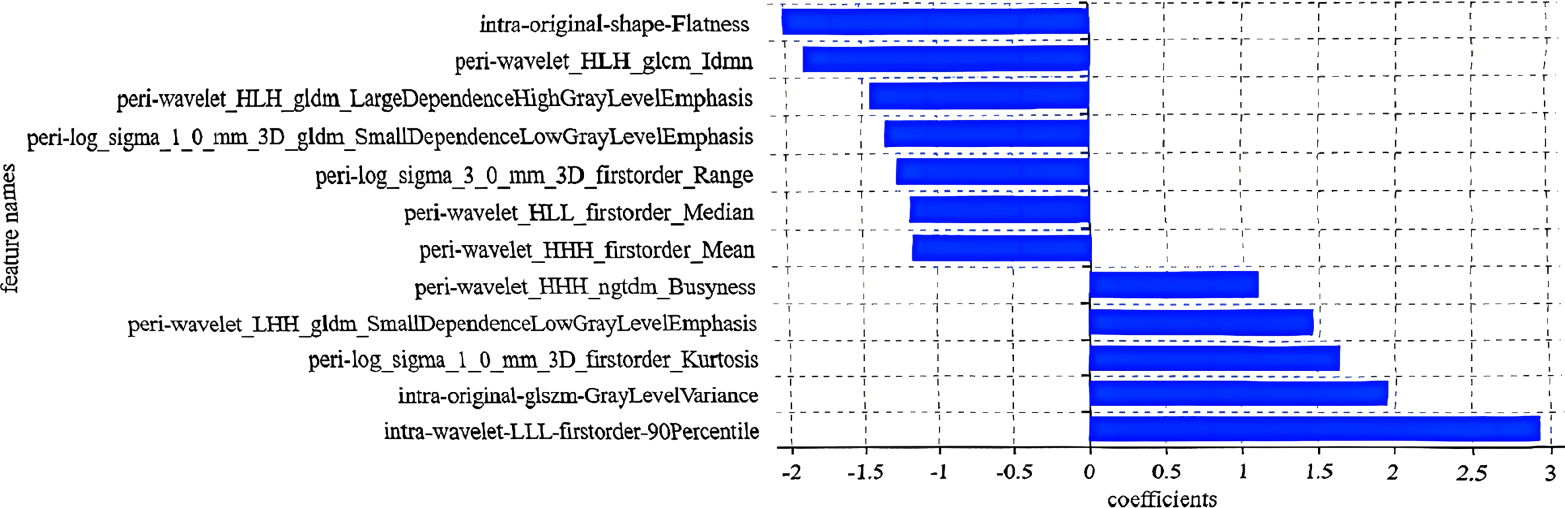
Radiomics features and coefficients of the combined radiomics model (*abscissa*: feature names; *ordinate*: coefficients).

After excluding those features with an ICC < 0.75, the remaining stable features were included in the subsequent analysis. For the intranodular, perinodular, and combined radiomics models, the optimal feature subsets consisted of 9, 13, and 12 features, respectively, which yielded the highest AUC values in the testing set. The selected features of all three models are presented in **Supplementary Table 1**, and the coefficients for the combined model are shown in **Figure 3**.

**Figure 4:**
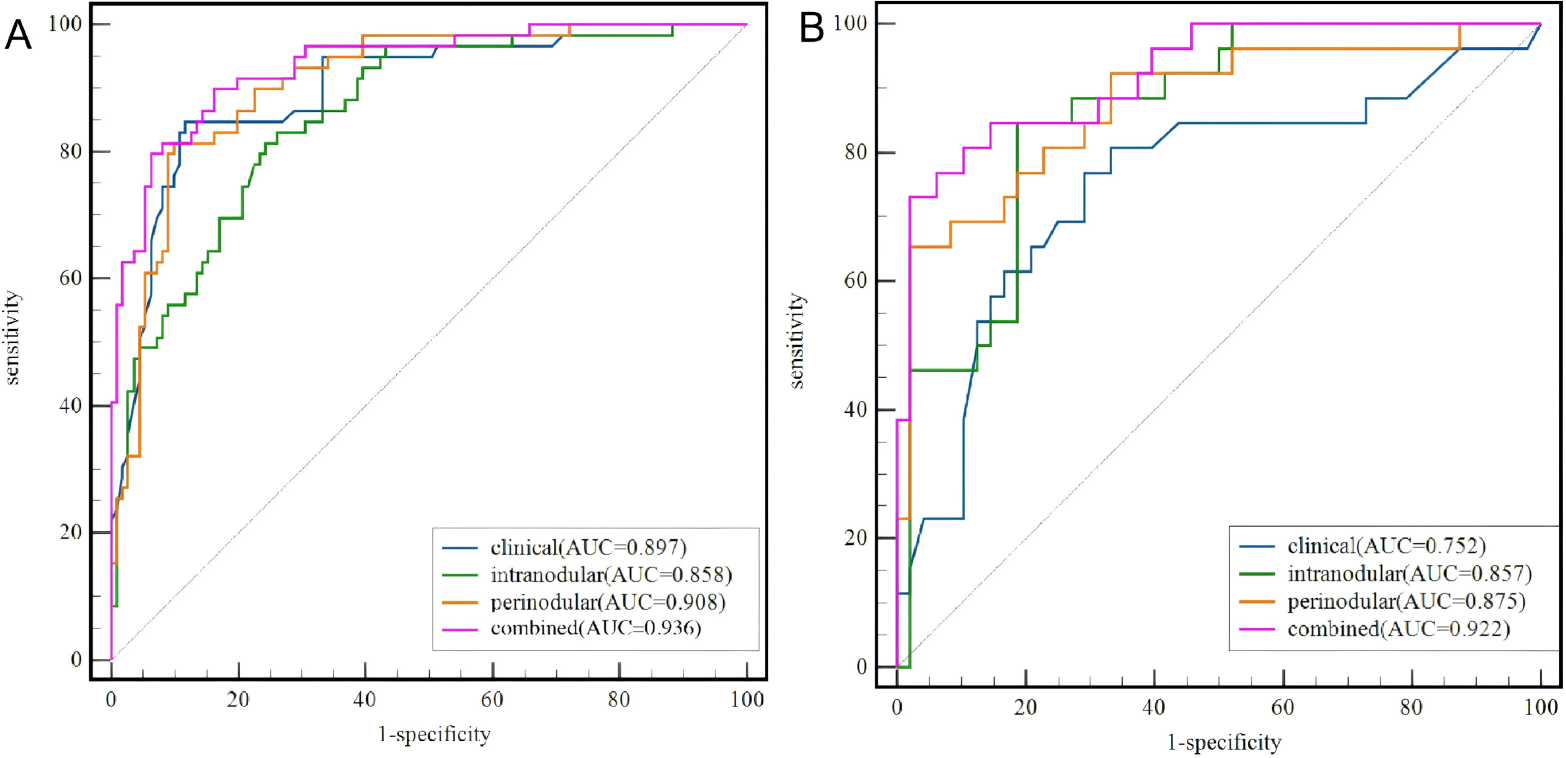
Receiver operating characteristic (ROC) curves of the clinical, intranodular, perinodular, and combined radiomics models in the training set **(A)** and the testing set **(B)**.

The AUC, sensitivity, specificity, accuracy, PPV, and NPV values of the clinical model and the three radiomics models are presented in **Table 3**. In the training set, the clinical model exhibited an AUC of 0.897 (95%CI = 0.841–0.938), with a sensitivity of 0.884, a specificity of 0.862, and an accuracy of 0.871. For the intranodular, perinodular, and combined radiomics models, the corresponding AUC values were 0.858 (95%CI = 0.801–0.915), 0.908 (95%CI = 0.862–0.953), and 0.936 (95%CI = 0.898–0.973), respectively. In the testing set, the clinical model showed an AUC of 0.752 (95%CI = 0.638–0.845), with a sensitivity of 0.739, a specificity of 0.786, and an accuracy of 0.750. The intranodular and perinodular models achieved AUCs of 0.857 (95%CI = 0.771–0.942) and 0.875 (95%CI = 0.786–0.964), respectively. Notably, the combined model attained the highest AUC of 0.922 (95%CI = 0.859–0.985), along with a sensitivity of 0.854, a specificity of 0.808, and an accuracy of 0.892. DeLong’s test analysis revealed that, in the training set, the combined model outperformed the intranodular model (*Z* = 3.736, *p* < 0.001). In the testing set, the combined model also demonstrated superior performance compared with the clinical model (*Z* = 2.808, *p* = 0.005). No statistically significant differences were observed among the other model comparisons. The receiver operating characteristics (ROC) curves and the detailed results of DeLong’s test are displayed in **Figure 4** and **Table 4**, respectively.

**Table 3:** Diagnostic performance of the clinical model and the three radiomics models.

Model	AUC (95%CI)	Specificity	Sensitivity	Accuracy	PPV	NPV
Training set						
Clinical	0.897 (0.841–0.938)	0.862	0.884	0.871	0.916	0.794
Intranodular	0.858 (0.801–0.915)	0.814	0.757	0.776	0.884	0.640
Perinodular	0.908 (0.862–0.953)	0.814	0.901	0.871	0.901	0.814
Combined	0.936 (0.898–0.973)	0.898	0.838	0.859	0.939	0.746
Testing set						
Clinical	0.752 (0.638–0.845)	0.786	0.739	0.750	0.850	0.590
Intranodular	0.857 (0.771–0.942)	0.808	0.812	0.824	0.907	0.710
Perinodular	0.875 (0.786–0.964)	0.769	0.813	0.865	0.839	0.944
Combined	0.922 (0.859–0.985)	0.808	0.854	0.892	0.870	0.950

*AUC*, area under the curve; *95%CI*, 95% confidence interval; *PPV*, positive predictive value; *NPV*, negative predictive value.

**Table 4:** Comparison of the areas under the curve (AUCs) of the clinical model and the three radiomics models.

Model	Training set		Testing set	
	*Z*-value	*p*-value	*Z*-value	*p*-value
Clinical *vs*. intranodular	0.931	0.352	1.541	0.123
Clinical *vs*. perinodular	0.314	0.753	1.643	0.100
Clinical *vs*. combined	1.131	0.258	2.808	0.005*
Intranodular *vs*. perinodular	1.649	0.099	0.330	0.742
Intranodular *vs*. combined	3.736	<0.001*	1.890	0.059
Perinodular *vs*. combined	1.612	0.107	1.175	0.240

* Indicates p < 0.05.

**Figure 5:**
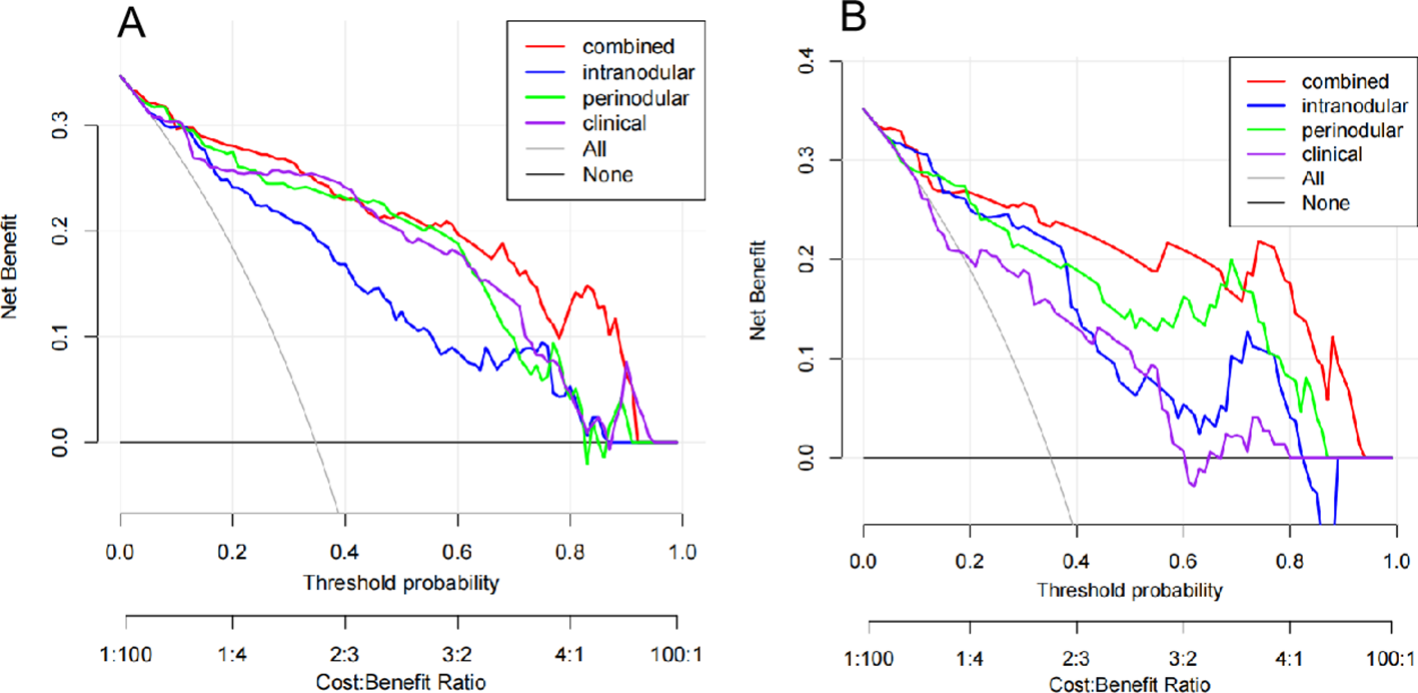
Decision curve analysis for the four models in the training set **(A)** and the testing set **(B)**. The *x*-axis represents the threshold probability. The *y*-axis represents the net benefit.

Furthermore, DCA (**Figure 5**) demonstrated that both the clinical model and the three radiomics models provided a relatively high overall net benefit in the training and testing sets. Specifically, in the testing set, the combined model achieved a higher overall net benefit than the other three models across reasonable threshold probability ranges, indicating its superior clinical utility in differentiating PC from LAC.

## Discussion

The purpose of this study was to investigate the utility of radiomics features derived from the intranodular and perinodular regions on non-contrast CT images for the noninvasive differentiation between PC and LAC. The results showed that both the intranodular and perinodular radiomics models demonstrated good discrimination in the training and testing sets. Notably, the combined radiomics model, which integrated features from both regions, outperformed the clinical model and the intranodular and perinodular models in accurately differentiating PC from LAC.

In clinical practice, radiologists currently rely primarily on the clinical and CT morphological characteristics for differentiation; however, this approach has substantial limitations. A multicenter study revealed that, when dependent on physicians’ experience, the average sensitivity, specificity, and accuracy in distinguishing PC from LAC were only 63.1%, 53.7%, and 59.3%, respectively (21). Several studies have revealed that morphological signs such as an irregular shape, lobulation, cavitation, necrosis, and mediastinal lymph node enlargement can also be observed in PC (22–24). In the present study, only age and pleural indentation sign differed significantly between PC and LAC, whereas lobulation, spiculation sign, and lesion location exhibited limited discriminative ability. As an inflammatory granulomatous disease, PC may mimic malignancies with spiculation or lobulation appearances due to inflammation and fibrosis. This imaging overlap reduces the specificity of conventional signs, accounting for their lack of independent predictive value in the clinical model. The clinical model achieved an AUC of 0.897 in the training set, but which decreased to 0.752 in the testing set, indicating relatively poor stability. This finding may be attributed to the subjective nature of the morphological feature evaluation and the inter-observer variability among radiologists. These limitations highlight the urgent need for more objective and biologically meaningful biomarkers to reliably differentiate these two diseases.

Radiomics has emerged as a powerful tool in medical imaging, enabling the extraction of quantitative features to link imaging phenotypes with the assessment of pulmonary nodules, molecular characterization, and prognostic evaluation (25–29). Leveraging this capability, numerous studies have investigated the discriminatory performance of radiomics signatures extracted from primary lesions in distinguishing granuloma from lung cancer (10, 15, 30–32). A recent meta-analysis confirmed the efficacy of CT-based radiomics in distinguishing tuberculosis from lung cancer, reporting a pooled sensitivity of 0.80, a specificity of 0.83, and an AUC of 0.88 (10). Similarly, studies by Zhao et al. (14) and Zhang et al. (15) indicated that intranodular radiomics signatures could distinguish PC from lung cancer, with AUCs exceeding 0.8. Consistent with these reports, the intranodular model in the present study achieved robust performance, with an AUC of 0.858 in the training set and a comparable AUC of 0.857 in the testing set. Although the diagnostic performance of the intranodular radiomics model was not superior to the clinical model in the training set, it exhibited better stability upon validation, underscoring the potential of radiomics to provide more generalizable biomarkers for this challenging differential diagnosis.

In recent years, considerable research attention has focused on the perinodular parenchyma, given its close association with the biological behavior of tumors such as invasiveness, metastatic potential, progression, and recurrence risk (17, 33, 34). In terms of differentiating between benign and malignant pulmonary nodules, several studies reported that radiomic features derived from the perinodular parenchyma can provide added value (35, 36). Beig et al. (17) extracted radiomics features from perinodular regions of varying widths (5–30 mm) to differentiate adenocarcinoma from benign pulmonary granulomas, finding that those from the 5-mm peritumoral region were the most prognostically informative. Masquelin et al. (37) reported that the 10-mm perinodular model achieved a higher AUC than the model based on the 15-mm perinodular zone for differentiating benign from malignant lung nodules. In contrast to using a fixed-distance perinodular region, Calheiros et al. (38) demonstrated that the combination of the features derived from a size-proportional perinodular zone (twice the nodule diameter) with intranodular features and margin sharpness features improved the diagnostic performance of models for solid pulmonary nodules. Similar to the study of Masquelin et al. (37), we extracted features from a 10-mm parenchyma region corresponding to the average secondary pulmonary lobule, which enabled capturing perinodular alterations at a biologically meaningful scale. Our results showed that the perinodular radiomics model achieved AUCs of 0.908 in the training set and 0.875 in the testing set, indicating predictive capability for distinguishing between PC and LAC. Despite this finding, there is no clear consensus with regard to the optimal extension range for the perinodular region across different studies, likely due to cohort heterogeneity and variability in the methodological robustness of feature extraction. Future studies should compare different margins in independent cohorts to identify the most robust configuration for clinical application.

We further integrated these extracted perinodular features with intranodular features to construct a combined model. The combined model achieved the highest AUC value in both the training and testing sets and demonstrated significantly superior performance compared with the clinical model in the independent testing set. Although the improvement in AUC over the single-region radiomics model did not reach statistical significance, which may be attributed to the relatively limited sample size, the DCA revealed that the combined model provided a greater net benefit than the other two radiomics models. The superior performance of the combined model can be attributed to its ability to integrate complementary information from both the primary lesion and the surrounding parenchyma. This integration enables more reliable patient stratification, potentially reducing unnecessary invasive procedures for patients with PC while ensuring timely interventions for those with LAC. Notably, the combined model exhibited a smaller decline in net benefit in the testing set. This indicates that the model was less prone to overfitting and possessed favorable generalizability, supporting its potential for clinical translation.

Practical considerations in the clinical application of radiomics must be addressed, in particular the segmentation variability, which remains an unavoidable and critical problem. As reported in previous studies, the inter-observer consistency in manual segmentation exhibits significant variability, which is influenced by multiple factors such as the nodule type, the nodule size, and the tissue types (39, 40). Subtle differences in boundary delineation may alter feature values, particularly for the texture and shape features that are sensitive to spatial definition. Moreover, segmentation-induced feature variability constitutes technical noise rather than biologically informative signals, which in turn impairs model robustness. In this study, image segmentation was performed by experienced senior radiologists, combined with multistep feature screening and dimensionality reduction, to ensure the robustness and stability of the extracted radiomics features. Despite the existence of segmentation variability, the radiomics model in this study still holds potential for the differential diagnosis of PC and LAC. However, the inherent subjectivity of manual segmentation cannot be fully eliminated. With technological advances, artificial intelligence-based radiomics enables automated image segmentation, minimizes manual intervention, and reduces segmentation variability (41). Therefore, future efforts should focus on the development of standardized automatic segmentation algorithms to reduce observer bias and ensure consistent feature extraction in multicenter settings.

Several limitations of our study should be acknowledged. Firstly, this study was designed as a retrospective investigation with only pathologically confirmed cases, which may have introduced selection bias. Secondly, inter-hospital variability in the CT protocols was mitigated through standardized image preprocessing; however, its potential influence cannot be entirely excluded. Thirdly, manual segmentation can introduce variability and affect model stability. To minimize this variability, all segmentations were performed by experienced senior radiologists. Future studies will explore automated segmentation algorithms to further reduce inter-observer variability. Fourthly, while our study included two centers with balanced distribution and strict dataset separation, the testing set was not entirely independent. Thus, external validation in a multicenter cohort is needed to confirm the generalizability of the model. Finally, the relatively modest sample size precluded robust subgroup analyses stratified by lesion size or morphological type (nodules *vs*. masses). Future studies with larger cohorts should incorporate these analyses to draw more comprehensive and reliable conclusions.

## Conclusions

In conclusion, by incorporating perinodular radiomics signatures, the combined model showed reliable diagnostic performance in differentiating between PC and LAC. The approach not only highlights the potential value of nodular surrounding parenchyma but also provides a more comprehensive quantitative tool for clinical decision support.

## Data Availability

The original contributions presented in the study are included in the article/**Supplementary Material**. Further inquiries can be directed to the corresponding author.
