# Supplementary material for: Intranodular and perinodular radiomics features based on non-contrast CT to distinguish pulmonary cryptococcosis from lung adenocarcinoma: a two-center study
Source: Front Oncol. 2026 Apr 27;16:1750773. doi: 10.3389/fonc.2026.1750773 (PMC13158070; doi:10.3389/fonc.2026.1750773)
Supplement: Supplementary file 1 [file Table1.docx]

**Table S1** Radiomics features and coefficients of the three models

| Feature name | Coefficient |
| --- | --- |
| Intranodular model |  |
| Original_shape_Flatness | -1.739382461 |
| Original_glszm_GrayLevelVariance | 1.716659003 |
| Log_sigma_1-0-mm_3D_glszm_SizeZoneNonUniformityNormalized | 0.856927128 |
| Wavelet_LLL_firstorder-90Percentile | 2.554448804 |
| Wavelet_HHL_glrlm_LongRunLowGrayLevelEmphasis | 1.533656725 |
| Wavelet_LHH_glszm_SmallAreaLowGrayLevelEmphasis | 1.332525334 |
| Wavelet_LLL_glszm_GrayLevelVariance | 0.773334981 |
| Wavelet_LHH_gldm_LargeDependenceHighGrayLevelEmphasis | -0.624295856 |
| Wavelet_HHH_gldm_LargeDependenceHighGrayLevelEmphasis | -0.907131208 |
| Perinodular model |  |
| original_shape_Elongation | -1.065477638 |
| Log_sigma_1_0_mm_3D_firstorder_Kurtosis | 3.598500314 |
| Log_sigma_1_0_mm_3D_gldm_SmallDependenceLowGrayLevelEmphasis | -1.865164632 |
| Log_sigma_1_0_mm_3D_ngtdm_Strength | 0.249349982 |
| Wavelet_HHH_firstorder_Skewness | 1.43528685 |
| Wavelet_HHH_glcm_Idn | -1.422721306 |
| Wavelet_HHH_gldm_DependenceVariance | -0.854986491 |
| Wavelet_HHL_firstorder_Mean | 1.053162319 |
| Wavelet_HHL_gldm_DependenceVariance | -0.348726554 |
| Wavelet_HLH_glcm_Idmn | -2.018238653 |
| Wavelet_HLL_firstorder_Median | -2.237136956 |
| Wavelet_LHH_gldm_SmallDependenceLowGrayLevelEmphasis | 1.754937165 |
| Wavelet_LHL_firstorder_Skewness | 0.86875257 |
| Combined model |  |
| intra-original_shape_Flatness | -2.001390478 |
| intra-original_glszm_GrayLevelVariance | 1.947860922 |
| intra-wavelet_LLL_firstorder-90Percentile | 2.927261992 |
| peri-log_sigma_1_0_mm_3D_firstorder_Kurtosis | 1.633038724 |
| peri-log_sigma_3_0_mm_3D_firstorder_Range | -1.268970708 |
| peri-log_sigma_1_0_mm_3D_gldm_SmallDependenceLowGrayLevelEmphasis | -1.348461224 |
| peri-wavelet_LHH_gldm_SmallDependenceLowGrayLevelEmphasis | 1.45227421 |
| peri-wavelet_HLH_gldm_LargeDependenceHighGrayLevelEmphasis | -1.44722551 |
| peri-wavelet_HLH_glcm_Idmn | -1.879524764 |
| peri-wavelet_HHH_ngtdm_Busyness | 1.094997565 |
| peri-wavelet_HHH_firstorder_Mean | -1.166599461 |
| peri-wavelet_HLL_firstorder_Median | -1.189023065 |

*GLSZM*, gray level size zone matrix; *GLRLM*, gray level run length matrix; *GLSZM*, grey level size zone matrix; *GLDM*, gray level dependence matrix; *GLCM*, grey level cooccurrence matrix
